# Encephalocraniocutaneous Lipomatosis Associated with Orbital Cyst: A Variant or New Entity?

**DOI:** 10.4274/tjo.galenos.2020.84584

**Published:** 2021-02-25

**Authors:** Abubakar Garba Farouk, Abubakar Farate, Zainab Yero Musa, Abba Bukar Zarami, Hajja-Falmata Kachallah Monguno

**Affiliations:** 1 University of Maiduguri College of Medical Sciences, Faculty of Clinical Sciences, Department of Pediatrics, Maiduguri, Nigeria; 2 University of Maiduguri Teaching Hospital, Department of Radiology,, Maiduguri, Nigeria; 3 University of Maiduguri Teaching Hospital, Department of Ophthalmology, Maiduguri, Nigeria; 4 University of Maiduguri College of Medical Sciences, Department of Histopathology, Faculty of Basic Clinical Sciences, Maiduguri, Nigeria; 5 University of Maiduguri Teaching Hospital, Department of Pediatrics, Maiduguri, Nigeria

**Keywords:** Encephalocraniocutaneous lipomatosis, choristoma, nevus psiloliparus, orbital cyst

## Abstract

Encephalocraniocutaneous lipomatosis (ECCL), also known as Haberland or Fishman syndrome, is an extremely rare congenital neurocutaneous disorder that characteristically involves ectomesodermal tissues such as the central nervous system, eyes, and skin. The etiology of the disease remains unknown. Here we present a rare case of ECCL associated with bilateral eye involvement and orbital cyst from Sub-Saharan Africa. A 3-year-old boy presented with cystic right eye swelling since birth. Physical examination showed alopecia on right side of the scalp, ipsilateral ocular cyst, and microphthalmia with a contralateral limbal dermoid. Computed tomography of the brain revealed severe atrophy of the right cerebral hemisphere with an expansion of the cerebrospinal fluid space and dilatation of the lateral ventricle suggesting ex-vacuo hydrocephalus. Right orbital cyst continuous with the globe and calcification of the posterior aspect of both globes were also present. Histopathologic findings of the excised orbital cyst revealed an eyeball covered by fatty tissue, calcification of the cyst wall, and corneal opacity. Microscopy showed cornea-sclera wall composed of normal cartilage communicating with sandwich bony trabeculae with a focus of marrow cells, consistent with choristoma. The constellation of these findings conforms to Moog’s revised diagnostic criteria for ECCL proposed in 2009. Although the disorder is easily recognizable at birth, neuroimaging is essential for appropriate diagnosis and management and to exclude or confirm other unusual associated abnormalities.

## Introduction

Encephalocraniocutaneous lipomatosis (ECCL), also known as Haberland or Fishman syndrome, is an extremely rare congenital neurocutaneous syndrome of unknown etiology, characterized primarily by cranial and facial asymmetry, cutaneous lesions, central nervous system (CNS) anomalies, and ocular abnormalities. It is a non-neoplastic disorder that was first described by Haberland and Perou in 1970.^1^ Nevus psiloliparus, a rare skin anomaly characterized by alopecia and excessive fat tissue is the hallmark of Haberland syndrome.^2^ The predominant ocular feature is eyelid choristoma but other ocular abnormalities include colobomas, corneal or anterior chamber abnormalities, and globe calcification. The CNS abnormalities are intracranial or intraspinal lipoma, abnormal intracranial vessels, complete or partial atrophy of a cerebral hemisphere, asymmetrically dilated ventricles or hydrocephalus, arachnoid cyst, porencephalic cyst, and calcification.^3^ Since the entity was described, several sporadic manifestations of the syndrome have been reported worldwide.^4,5,6,7^ The syndrome does not have clear gender preponderance and no known racial or geographical predilection.The manifestations of ECCL resemble other congenital developmental disorders such as oculocerebrocutaneous syndrome, linear nevus sebaceous syndrome, Goltz syndrome, and Goldenhar syndrome. We present the case of a 3-year-old boy with classical clinical and neuroimaging features of ECCL and an unusual orbital cyst, a finding that has not previously been described in this syndrome to the best of authors’ knowledge.

## Case Report

A 3-year-old boy was noticed to have a swelling in place of the right eye at birth that was about the size of the pulp of the thumb and progressively increased to about the size of a lemon as the child was growing. At the same time of birth, a brownish fleshy mass was also seen in the left eye which remained stable without appreciable change in size over time and he was said to be seeing with the eye. He was also noticed to have dark coloration on the right side of the head with relatively faster growth of the right side of the head. He had no history suggestive of hearing impairment and he had never convulsed. His mother had no exposure to ionizing radiation during pregnancy and no previous delivery of a congenitally malformed baby. Although he had normal development, he appeared to have delay compared to his siblings. He had no cardiopulmonary symptoms and no limb or joint deformities.

**Figure 1:**
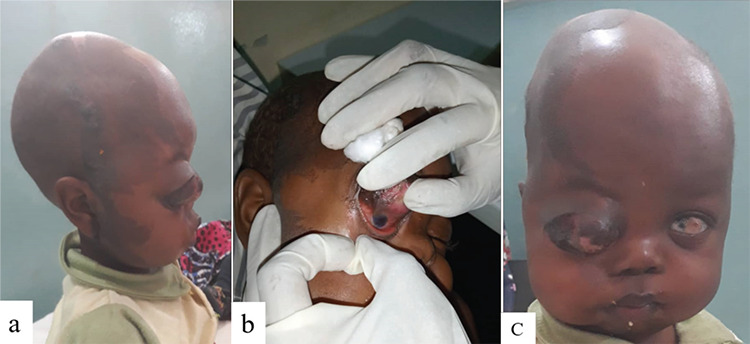
Clinical photographs of the patient: (a) flaky granular pigmented hairless lesion over the right frontotemporal region extending down to the cheek; (b) right cystic eye with microphthalmia, and (c) craniofacial asymmetry, right orbital mass, and left eye limbal dermoid

Physical examination revealed a small-for-age looking child with an obvious asymmetry of the head; the right side was larger than the left around the parietal, parieto-temporal, and frontal regions. A hyperpigmented hairless skin lesion was noted over the hypertrophied right parieto-temporal and frontal regions of head extending down the pre-auricular area to the cheek in an irregular fashion (Figure 1a). His head circumference was within normal limits (47.5 cm, 10^th^ percentile for age).

CNS examination revealed a conscious and alert child with grossly intact cranial nerves and no focal neurologic deficits. There was a cystic non-tender right orbital mass measuring 6x5x4 cm, covered by hyperpigmented skin and attached to the upper eyelid. Beneath and continuous with the right eye mass was a small globe (microphthalmia) with cornea measuring 4 mm vertically and 5 mm horizontally, partial aniridia superiorly, no light perception, and normal lower eyelid (Figure 1b). He fixated to light and reached out for objects seen with the left eye, which had inferior temporal limbal dermoid, normal pupil, and was reactive to light (Figure 1c). Other systemic examinations including the cardiovascular system were essentially normal. Hematologic profile was within normal limits. 

Ultrasound scan of the right orbit showed a huge thick-walled cystic lesion with multiple strands and internal echoes. The anterior and posterior chambers, as well as the vitreous humour, could not be distinctly identified. 

**Figure 2:**
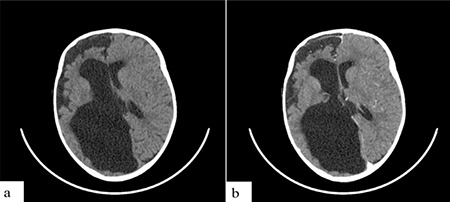
Axial non-contrast-enhanced (a) and contrast-enhanced (b) computed tomography of the brain showing severe atrophy of the right cerebral hemisphere with an expansion of the cerebrospinal fluid space over the ipsilateral cerebral convexity and dilatation of the lateral ventricle on the same side (the so-called “exvacuo hydrocephalus”). No areas of abnormal contrast enhancement

**Figure 3:**
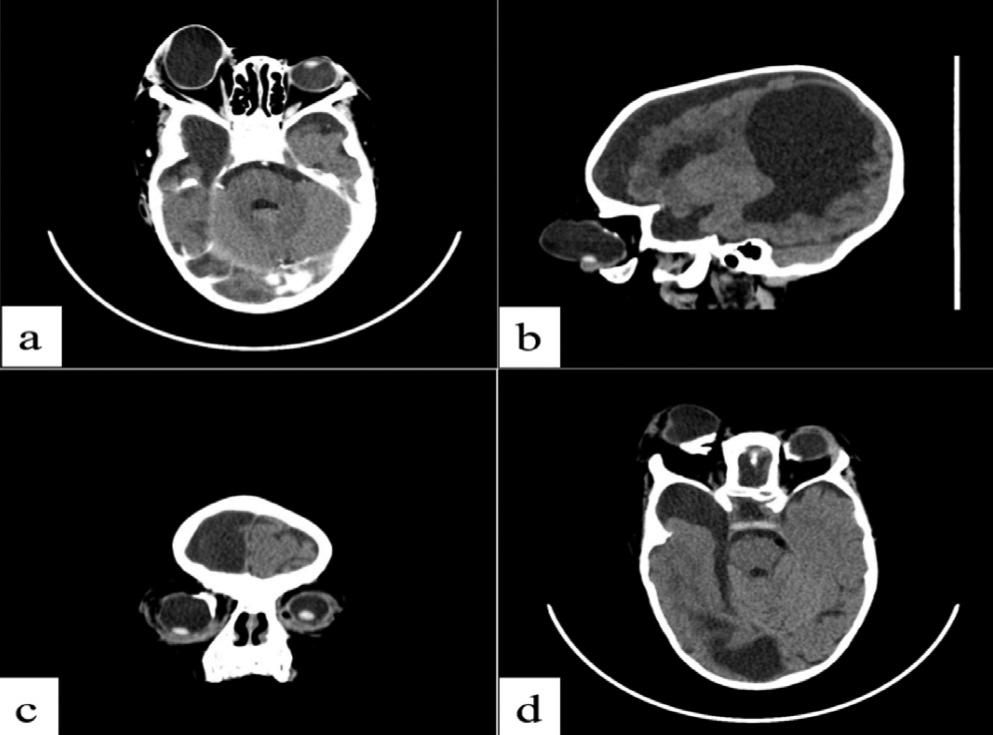
Axial computed tomography of the brain at the level of the orbit showing a cystic right orbital mass on the same side of the cerebral lesion with inferior displacement of the lens of the eye (a-c) and associated calcification of the posterior aspect of the globes of both eyes (d)

Computed tomography of the brain revealed severe atrophy of the right cerebral hemisphere with an expansion of the cerebrospinal fluid space over the ipsilateral cerebral convexity and dilatation of the lateral ventricle on the same side, suggesting ex-vacuo hydrocephalus (Figure 2). There were no intracranial masses or areas of abnormal enhancement after intravenous injection of contrast medium. A cystic right orbital mass on the same side of the cerebral lesion with inferior displacement of the lens and bilateral calcification of the posterior aspect of the globes were also seen (Figure 3). 

A modified enucleation of the right eye was done under general anesthesia. The right upper eyelid was separated from the mass by blunt dissection. The mass was well-circumscribed and continuous with the globe. The specimen was sent immediately to the laboratory unfixed for histopathological analysis.

**Figure 4:**
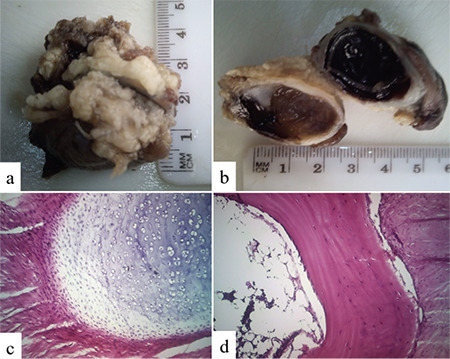
Macroscopic view of the right eyeball covered by periorbital fat (a) with the cut surface of the eyeball revealing a thick calcified wall and corneal opacity (b). Photomicrographs (c and d) show cornea-sclera wall within which are normal cartilage that is communicating with a sandwich bony trabeculae with a focus of marrow cells (hematoxylin and eosin, x100)

Histopathologically, the findings on macroscopy were an eyeball covered by periorbital fat, measuring 5x4x3 cm and the cut surface showed a calcified wall with corneal opacity (Figure 4a, b). Microscopy showed cornea-sclera wall composed of normal cartilage communicating with sandwich bony trabeculae with a focus of marrow cells, consistent with choristoma (Figure 4c, d). 

## Discussion

Encephalocraniocutaneous lipomatosis was first described by Haberland and Perou in 1970,^1^ and therefore, the syndrome was named after Haberland. Subsequently, Fishman in 1987 reported 3 additional cases. Hence, it was also termed Fishman syndrome.^4,5,6^ There is no clear gender, racial, or geographical predilection of this disorder.^3,8,9^ The pathogenesis of ECCL remains unclear. There is no evidence suggestive of genetic inheritance or chromosomal abnormality. Dysgenesis of the cephalic neural crest and the anterior neural tube, suggested by Haberland in 1970,^1^ remains the most widely accepted theory of pathogenesis in favor of somatic mosaicism, which is thought to be the underlying pathophysiology in ECCL.

The hairless lesion of the scalp (nevus psiloliparus) is pathognomonic and a dominant component of the syndrome, especially when it is accompanied by excessive deposition of fatty tissue.^2^ The predominant ocular feature is eyelid choristoma but other ocular abnormalities include colobomas, corneal or anterior chamber abnormalities and globe calcification. CNS abnormalities are intracranial or intraspinal lipoma, abnormal intracranial vessels, complete or partial atrophy of a cerebral hemisphere, asymmetrically dilated ventricles or hydrocephalus, arachnoid cyst, porencephalic cyst, and intracranial calcification.^1,2,3,7,10^ Further features that characterize the syndrome are cranial and facial asymmetry, hamartoma, and occasionally visceral lipomas. The patient in this report had the classical triad of systemic involvement and fulfilled the Moog’s revised diagnostic criteria for the diagnosis of ECCL.^3^ He had the following clinical and neuroimaging features that are consistent with the syndrome: cranial and facial asymmetry, streaky non-scarring alopecia, ocular choristoma, asymmetrically dilated ventricles, atrophy of the right cerebral hemisphere, and bilateral globe calcifications, all of which are well documented in the previous literature.^1,2,3,4,5,6,7^ However, the unusual aspect of this case was that the right orbital cyst was continuous with the globe, a finding that has not been documented in ECCL. The orbital cyst is one of the prominent features in oculocerebrocutaneous (Delleman-Oorthuys) syndrome. Thus, it is unclear whether this represents an overlap between the two neurocutaneous syndromes or perhaps a new entity that has not been documented in previous literature to the best of the authors’ knowledge.

The anomalies in ECCL tend to be unilateral, although bilateral eye involvement as seen in our patient has been described.^11,12^ Other clinical findings associated with ECCL are seizures and mental retardation ^5^, neither of which were found in our patient. The absence of neurologic deficits despite the degree of intracranial malformations detected on neuroimaging is similar to previously reported cases where the observed neurologic deficits were less severe than the intracranial abnormalities.^3,10^ Nevertheless, long-term follow-up is necessary, as the child’s neurologic status may deteriorate as he grows older.

The differential diagnosis of ECCL includes oculocerebrocutaneous (Delleman-Oorthuys), syndrome, Sturge-Weber, Proteus syndrome, nevus sebaceous syndrome, oculo-auriculo-vertebral (Goldenhar) syndrome, and oculoectodermal syndrome, and potential overlap between these neurocutaneous disorders may cause difficulty in establishing a diagnosis.^7,10,13,14,15^ A good example is this case, where the patient presented typical features of ECCL but also had an unusual orbital cyst, a feature that is commonly encountered in Delleman-Oorthuys syndrome.

The treatment of ocular lesions is mainly cosmetic and includes excision of conjunctival tumors and lamellar or penetrating keratoplasty.^16^ Our patient underwent a modified enucleation of the cystic right eye. Dysmorphic cutaneous lesions in this disorder may require surgical treatment. Most patients with ECCL have normal development despite CNS changes while a few may present with severe mental retardation.^3^

To the best of our knowledge, this is the first case in our collective experience in which a patient presented with characteristic multiple organ involvement typical of ECCL associated with an orbital cyst. Orbital cyst is commonly associated with oculocerebrocutaneous (Delleman-Oorthuys) syndrome. It is unclear whether this represents an overlap between the two neurocutaneous syndromes or perhaps a new entity that has not been documented in the previous literature. Neuroimaging is essential for appropriate diagnosis and management of ECCL and to exclude or confirm other unusual associated abnormalities, as in this case. Parents of a child with ECCL should also be reassured that the risk of occurrence in siblings is virtually absent and the genetic syndrome is not transmittable to offspring.
